# Laboratory considerations amidst the coronavirus disease 2019 outbreak: the Spedali Civili in Brescia experience

**DOI:** 10.4155/bio-2020-0109

**Published:** 2020-06-23

**Authors:** Emirena Garrafa, Duilio Brugnoni, Mosè Barbaro, Laura Andreoli, Emanuele Focà, Massimo Salvetti, Francesco Castelli, Franco Franceschini, Simone Piva, Maria Lorenza Muiesan, Nicola Latronico, Rosella Levaggi

**Affiliations:** ^1^Department of Clinical & Experimental Sciences, University of Brescia, Brescia, Italy; ^2^Department of Laboratory Diagnostics, ASST Spedali Civili, Brescia, Italy; ^3^Unit of Rheumatology & Clinical Immunology, ASST Spedali Civili, Brescia, Italy; ^4^Department of Clinical & Experimental Sciences, University of Brescia, Brescia, Italy; ^5^University Division of Infectious & Tropical Diseases, ASST Spedali Civili, Brescia, Italy; ^6^2nd Medicine Unit, ASST Spedali Civili, Brescia, Italy; ^7^Department of Anesthesia, Critical Care and Emergency, Spedali Civili University Hospital, Brescia, Italy; ^8^Department of Medical & Surgical Specialties, Radiological Sciences & Public Health, University of Brescia, Brescia, Italy; ^9^Department of Economics & Management, University of Brescia, Brescia, Italy

**Keywords:** COVID-19 emergency, COVID hospital, lab organization, panels for monitoring patients, reagents, staff management

## Abstract

Coronavirus disease 2019 emergency has created an enormous stress on providers that have been transformed into coronavirus disease hospitals. This article presents the experience of the clinical laboratory of Spedali Civili in Brescia (a teaching hospital in Lombardy with over 1500 beds) in managing the crisis, and to offer practical considerations for laboratory testing for this cohort of patients. Our contribution is threefold: by comparing the demand for tests in two representative period before and within the crisis, we show the change in compositions of the analytes that other labs may expect; we present the new panels of tests that hospital staff can order with different advantage for wards and laboratory; and we show how to reorganize staff on the basis of changes mentioned above.

Coronavirus disease 2019 (COVID-19), is a disease sustained by a virus belonging to the Coronavirus family, denominated as SARS-CoV-2, originated from the town of Wuhan in China [1], which has rapidly expanded from China worldwide.

In Italy, the first official registered case was reported on 20 February; since then the virus has spread very quickly across the country, in particular in Lombardy, a region in the north western part of Italy with a population of about 10 million inhabitants. Brescia (a province in east Lombardy that accounts for 1,200,000 inhabitants), rapidly become one of the most affected area [2].

The central laboratory of Spedali Civili, Brescia is one of the biggest in Lombardy. It performs test for the inpatients of Spedali Civili, a large university hospital with 1570 beds, and for outpatients, serving an area of nearly one million people in the east of Lombardy.

The central laboratory consists of various departments such as clinical chemistry, hematology, immunology, toxicology, urinalysis and clinical immunology to provide the tests required by hospital wards and outpatients. It does not perform microbiological analysis that have a dedicated laboratory.

The technical staff consists of almost 56 technicians that are distributed among the different activities. Thirty of them are assigned to the activities that converge in the core lab and guarantee their presence 24 h; the others are distributed on the specialist areas and work on shifts 5/6 days a week. Twenty physicians complete the team; they supervise specific laboratory sector, and at least one of them should always be in the lab to deal with emergencies. In our laboratory, we use several methodologies for testing samples, from manual to fully automated tests, both for highly demanded and less required analytes. The increasing complexity of lab procedure (methodologies, technology and troubleshooting equipment), requires to employ highly specialized staff; hence, there is a learning cost in moving staff across production lines.

Before the outbreak of COVID-19 emergency, the number of requests for tests to be performed in the lab was around 500.000 per month. Among all the tests, 48% were requested for inpatients in different wards (pulmonology, infectious diseases, pediatrics, surgery, maternity, internal medicine, etc.) while the remaining were performed for outpatients and are prescribed either by general practitioners or by specialist. These tests are mainly devoted to screening, routine checkups for chronic patients, follow-up and pre-operative screening. The laboratory is also a reference point for the clinical tests that other laboratories do not perform. Being a reference lab for the province implies that some of the tests performed in our laboratory are highly frequent, while others are specialist exams seldom demanded. The great majority of the tests are performed on the core lab, a total laboratory automation (TLA), where almost every step of the laboratory process is automated. The other tests are performed in a different area of the laboratory, where special expertise and specialized equipment are kept. This appeared to be, before the outbreak of COVID-19 infection, the best organization to process the request of tests from our clinicians and from the territory. In fact, the great majority of our tests respect the turnaround time [3]. However, since the first COVID-19 patient was admitted on 23 February, Spedali Civili of Brescia has rapidly become overloaded with patients with pneumonia and severe acute respiratory distress syndrome. More than 1000 patients were admitted for this pathology, and at present, about 600 are the inpatients with COVID-19, 48 of which are in intensive care unit (ICU), a number in line with what has been observed in the literature [2]. Our hospital has rapidly changed its clinical priority and the demand for clinical tests with important change not only for the wards but also for the laboratory [2]. This has meant a rapid change in the organization of the production lines, reagents restocking and a change in the staff duties. In this paper, we would like to offer our experience on how laboratories may contribute to cope with the emergency by supporting medical staff, both as concerns meeting the new demand without reducing the usual quality standard, and also by reorganizing its staff across production lines. The former objective is reached by a continuous collaboration and exchange with our clinicians and with the laboratory staff. The literature has already pointed out the importance of labs in the management of this crisis [4,5], and our contribution adds a pragmatic point of view, highlighting how demand dramatically changed and how a laboratory can respond to this sudden switch.

## The change in demand

The COVID-19 crisis has significantly changed the demand for clinical tests. In spite of the actions to reduce the risk of contagion, most outpatients have postponed routine tests and specialist visits, while the hospital has cancelled nonurgent services (such as programmed surgery, day hospital for most chronic illnesses, outpatients clinics, etc.) meaning that some tests were no longer prescribed. For the laboratory, this has meant a sharp reduction in the demand for tests and a change in its composition. To show this, we compared the requests for two representative periods: 24 February–24 March 2020, in the middle of COVID-19 epidemic; and the same period in 2019, when no epidemic or other health problems were recorded. The tests processed were well balanced between outpatients and inpatients in 2019, while, during the COVID-19 outbreak, the demand for tests from outpatients almost disappeared probably because of the actions that have been put in place to reduce the risk of contagion such as a government decree that essentially prohibited all movements of people within the whole territory. The demand for inpatients was rather stable because, as mentioned before, the hospital became a reference point for patients with pneumonia and severe acute respiratory distress syndrome consequent to SARS-CoV-2.

**Figure 1.**
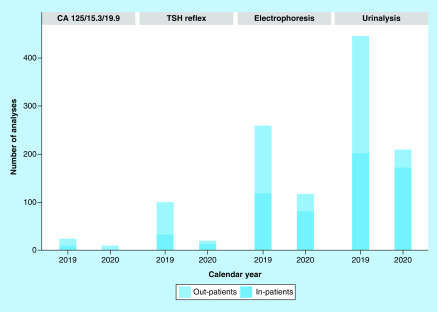
Change in the composition of the demand for less requested tests outbreak for inpatients and outpatients.

For the internal organization of the lab, it is even more important to examine the change in the composition of the tests requested, which was substantial when comparing the requests for two representative days: Monday, 16 March 2019 and Monday, 18 March 2020, in the middle of COVID-19 outbreak. Figure 1 presents the change in the number and the composition of the demand for lab tests.

As shown in Figure 1, the demand for some tests almost disappeared overnight: for example, tests whose demand is mainly generated by follow-up of patients (carbohidrat antigen [CA] 125, CA 19.9 and CA 15.5 for tumor markers, and tyroid stimulating hormon [TSH] as representative of hormone). The same pattern presented in Figure 1 has been observed for test devoted to autoimmunity, toxicology and allergology (data not shown). For some tests such as electrophoresis and urinalysis, the demand was rather stable for inpatients, but almost vanished for outpatients. Most of these are routine tests for chronically ill patients and we expect a sharp and anomalous increase as soon as the COVID-19 emergency will be over.

**Figure 2.**
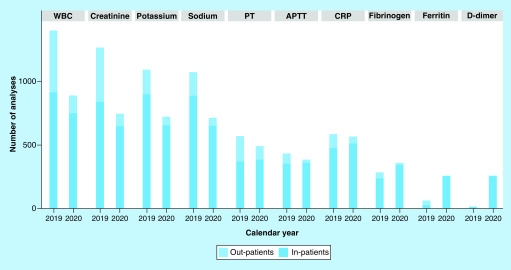
Change in the composition of the demand for highly requested tests outbreak for inpatients and outpatients.

The pattern for highly requested test has changed: creatinine, white blood cell (WBC), potassium have experienced a sharp decrease, only few of them have experienced a stable demand. On the other hand, as shown in Figure 2, the demand of some tests such as D-dimer, fibrinogen and ferritin have dramatically increased. These tests, together with prothrombine time (PT), activated partial thomboplastin time (APTT), CRP, blood count, glucose, creatinine, aspartate amminotrasferas (AST), alanine amminotrsaferase (ALT), gamma-glutamyl transferase (γ-Gt), triglycerides, sodium, potassium, calcium, lactate dehydrogenase (LDH) are the most requested for COVID-19 patients from all the wards, because they are useful to clinically frame these patients and to detect hyperinflammation and thrombotic events that seems to be common in these patients [6–8].

Most of the tests required by our clinician had already a dedicated ‘line of production’ in our lab. Other tests, such as IL-6 (preliminary data from China suggested to use it to monitor patients under treatment with tocilizumab [9]), were added to the offer of the laboratory without affecting the usual quality standard. In the first days of the pandemic, only few therapeutic approaches existed for treating COVID-19 patients. Other therapeutic approaches were developed later, and we are currently using a wider range of tests, but for a reduced number of patients since the number of new cases has decreased. In this emergency, the choice of the platform to be used becomes important to secure the quality process. We have chosen TLA to process new test for different reasons:

- Automation is the safest alternative for managing infected material. Our TLA installation combines several instruments and work cells with a specimen management and transportation system as well as a process control software component to automate a large percentage of laboratory work including centrifuge, decapping, sorting of samples, testing on various instruments, inspection, post analytical processing, storage and retrieval;
- TLA is operative 24 h every day, while other areas of the laboratory operate 5 days/week;
- We can use a single tube for different analytes, thus avoiding blood sample transport, manipulation and discharging of extra tubes with infected material. Furthermore, for tests performed using the TLA, the automatic storage of the blood sample allows to perform additional tests (if required by the medical staff) without adding a new blood drawing offering timely of results;
- There is no need of extra staff for additional lines: the running costs incurred are simply reagents and cleaning materials.

## New panels of test as a support to the medical staff

The process of converting medical, surgical and other wards into COVID-19 division has meant that the demand of clinical labs is now concentrated on specific batches of tests that usually reflects the different progression of the disease.

In order to reduce the time spent by the medical staff to list the tests requested, each ward has defined batteries of routine basic tests (defined alternatively as panels or profiles) for diagnosis and treatment. This opportunity was built in order to allow a quick prescription of tests needed as well as to reduce the entry into the high-isolation rooms (or ward) of the nursing staff to perform blood sampling. They can be ordered by a click of mouse without selecting every single test leaving the opportunity to add any additional test needed for clinical practice. The composition of the panel reflects local, regional or national practices and local ward level. Moreover, the panel has been approved by all the clinicians involved in the COVID-19 patients’ care: intensive care and infectious diseases specialists, pulmonologists, internal medicine specialists and immune rheumatologists.

After the outbreak in China, Lombardy was the first region in Europe to be hit by this emergency. The process of converting medical, surgical and other wards into COVID-19 divisions has meant that the demand of clinical labs had to be concentrated on specific batches of tests that reflect the different progression of the disease as shown above. Few suggestion on which biomarker should be used for COVID-19 patients are reported in the literature but nothing ‘consolidated’ exists [1,5,7,8]. Furthermore, none of pre-existent panels offered by our laboratory was appropriate for COVID-19 patients because they were either redundant or because they did not include any specific markers. In agreement with clinicians, we set up new panels that reflect the need of the COVID wards, confirming that collaboration between laboratories, clinicians and other healthcare professionals is crucial [10].

We set up two types of panels: ‘medical wards panels’ and ICU panels (see Table 1). For medical wards, we set up ‘COVID admission’, which is appropriate for patients just admitted to the hospital. The panel includes: blood count; glucose; creatinine; CRP, AST, ALT, γ-Gt, triglycerides, sodium, potassium, calcium, LDH, PT-INR, APTT, fibrinogen, D-dimer, ferritin, CPK, troponin, protein electrophoresis and urinalysis. ‘COVID routine’, a panel which excludes troponin, protein electrophoresis and urinalysis from the list above, is used for monitoring patients.

**Table 1.** Panels of analytes set up for request by the intensive care unit and by nonintensive care unit wards.

Panel name	Panels for non-ICU wards		Panels for ICU							
	COVID admission	COVID monitor	COVID-ICU admission	COVID-ICU Monday	COVID-ICU Tuesday	COVID-ICU Wednesday	COVID-ICU Thursday	COVID-ICU Friday	COVID-ICU Saturday	COVID-ICU Sunday
Albumin			X		X			X		
ALP			X		X			X		
ALT	X									
APTT	X	X	X	X		X		X		
AST	X									
Blood cell count	X	X	X	X	X	X	X	X	X	X
Calcium	X	X	X		X			X		
Chlorine			X		X			X		
Cholesterol-total			X		X			X		
CK	X									
Creatinine	X	X	X	X		X	X	X	X	X
Creatinine clearance					X					
CRP	X			X		X	X		X	X
D-dimer	X	X	X	X		X		X		
Electrophoresis	X									
Ferritin	X	X	X	X		X		X		
Fibrinogen	X	X	X	X		X		X		
Formula	X	X	X	X		X			X	
GGT	X	X	X		X			X		
Glucose	X	X	X		X			X		
Ig (IgA, IgM, IgG)			X							
LDH	X	X	X		X			X		
Magnesium			X		X			X		
NT-PROBNP			X							
Phosphorus			X		X			X		
Potassium	X	X	X	X	X	X	X	X	X	X
PT-INR	X	X	X	X		X		X		
Sodium	X	X	X	X	X	X	X	X	X	X
Total bilirubin		X	X		X			X		
Triglycerides	X	X	X		X			X		
Troponin	X		X		X			X		
Urea			X		X			X		
Uric acid			X		X			X		
Urinalysis	X		X	X			X			

ALP: Alkaline phosphatase; ALT: Alanine aminotransferase; APTT: Activated partial thromboplastine time; AST: Aspartate aminotrasferase; CK: Creatine kinase; COVID: Coronavirus disease; GGT: Gamma glutamyl trasferase; ICU: Intensive care unit; Ig: Immunoglobulin; INR: International normalized ratio; LDH: Lactate dehidrogenase; NT-PROBNP: N terminal fragment of brain natriuretic peptide; PT-INR: Phrotrombine time-international normalized ratio.

Setting panels for the ICU was more complicated because these patients are more severe, their vital parameters less stabel, and need to be monitored daily. For this reason, we set a day-specific panel for ICU admission and a weekly panel that allows rotating the tests that are needed to follow the progress of the patients. The panels include the analyte in Table 1 and can be ordered by a click of mouse.

Panels mean that at any time, any physician, in any ward of the hospital knows that by flagging on a specific profile they will receive in few hours all the tests required to treat the patient. This is very important because most COVID-19 units are run at present by physicians dealing with an enormous unexpected problem, the turnover in the ward is very high, shifts are long, demanding and also the medical staff should avoid as much as possible strict contact with other colleagues.

For the laboratory, this also represents an advantage: the definition of the panel allows an accurate forecast of the quantity of reagents needed. In a period where provision is becoming difficult, we avoid the problem of running short of essential reagents. Furthermore, we avoid unnecessary manipulation of contaminated material when wards were asking some extra (forgotten) analytes. Panels set up with the collaboration of clinicians allows to increase vallue for money because, by listing only appropriate tests, they allow to reduce costs. In a time of pandemic where restocking is more difficult, they also allow to reduce the risk of running of reagents. Another strategy we adopted was to manage the priority of this panel versus routine exams. This is an important point since the turnover of the patients, due to admission, transfer to ICU and death, does not justify the request of the laboratory to ask exams mainly in the morning.

## Staff management

The laboratory has also undergone a revolutionary internal reorganization. We always adopted WHO guidelines for sample manipulation which has reduced the risk of staff getting infected, during working hours, However, due to the widespread of the disease among the population, staff got infected outside the laboratory. At the time of writing, 10% of the staff (four physicians and eight technicians) was found positive to COVID-19. Positive staff should be replaced immediately, for a variable time depending on being simply positive (quarantine of at least 2 weeks) or being also symptomatic (a longer period). Within the laboratory, due to the specific skills of the workforce, this is not easy to be carried out (due the nature of the infection, the staff of the same laboratory section is likely to become ill at the same time). In our experience, it may be important to invest in cross lab training: in the case of emergency as many workers as possible do have the skills (especially from a technical point of view) required to manage the most requested exams.

## Conclusion

In this paper, we presented our experience in order to give some pragmatic suggestions regarding ways to tackle the emergency. Information exchange with clinicians helps the clinical and the laboratory staff to understand which priorities are to be set. The organisation of work in the lab should be reorganized both to respond to the change in demand and to avoid shortages of essential reagent material, which would affect the usual quality standards. In the unlikely event of a second COVID-19 outbreak, the panels could be quickly retrieved so that the hospital could be transformed once again into a COVID hospital.

While we hope that the current emergency will end quickly, we are aware that, as soon as the pandemic is over, we will have to be ready to manage the flow of routine testing that has been postponed in its wake. In the next few weeks new staff will be hired, allowing us to increase the number of tests that can be performed, and to secure cross-training among platforms.

## Future perspective

Clinical laboratories represent an area of healthcare that has always undergone major changes because of technological advances, relations with wards and economical pressure. The outbreak of COVID-19 has obliged a quick reorganization that has shown the advantages and limitations, but also the potential, of the actual organization model.

Most of the tests for COVID were performed on TLA, so we simply needed to implement training for new test on staff already able to manage automation, and this did not affect work overtime.

Thanks to TLA installations, we could manage infected material without additional risk, we could easily add new tests required for this pathology, we could supply most of the tests required 24 h with the usual quality standard in spite of the rapid change in demand. However, the lack of sufficient staff cross-specialization has meant that staff devoted to the tests, whose demand has dramatically dropped, could not be used to increase the supply of the tests required during this pandemic event. The future perspective is then to increase automation where possible and to retrain internal staff across specialized fields. We are training new staff to work on TLA so that, in case of a new wave of infection, we will have more staff able to deal with this kind of exams. We will also implement training for less requested exams because as soon as the pandemic wave disappears we will have to face the wave of exams of chronic condition that were not required in these months.

The hospital management has decided to hire new staff to increase the capacity of our lab; this will allow us to perform more tests and to start the cross-training program, that this pandemic event has proved to be so vital.

Now, that things are going better and we have more time, we are training new staff to work on TLA so in case of a new wave of infection we will have more staff able to deal with this kind of exams. We will also implement training for less requested exams because as soon as the pandemic wave disappears we will have to face the wave of exams of chronic condition that were not required in these months. The hospital managers have decided to hire new staff so it will be more easier to train the staff without affecting the daily work

Finally, due to the emergency, the collaboration with the medical staff involved in the ward has proved vital to respond quickly to the crisis with a look to economic costs; this is a value of the organization model of our lab that we need to keep vital.

**Executive summary**

- Coronavirus disease 2019 (COVID-19) emergency has created an enormous stress on regional healthcare systems in Italy at different levels. This is especially true for hospitals that have been transformed into COVID hospitals almost overnight.
- The aim of this article is to present the experience of the clinical laboratory of Spedali Civili in Brescia (a teaching hospital in Lombardy with more than 1500 beds) in the management of the emerging crisis and to offer practical considerations for different aspects of laboratory testing in this cohort of patients.
- The unexpected change in the demand for tests has required a complete reorganization of the laboratory: a sudden modification in the distribution of reagents on the production lines, restocking of reagents that were seldom used before the crisis and a reorganization of the laboratory staff. Our contribution is threefold:
  - By comparing the demand for tests in two representative periods, before and within the crisis outbreak, we show change in compositions of the analytes that other labs may not expect. The pattern for highly requested test has changed: creatinine, WBC, and potassium have experienced a sharp decrease. On the other hand, the demand for D-dimer, fibrinogen and ferritin has dramatically increased. These tests, together with PT, APTT, CRP, blood count, glucose, creatinine, AST, ALT, γ-Gt, triglycerides, sodium, potassium, calcium and LDH are the most requested for COVID-19 patients from all the wards;
  - We present the new panels of tests that were set and that the hospital staff can order, with different advantages for wards and laboratories. We have set up two types of panels: medical wards panels and intensive care unit panels (Table 1). They can be ordered by a click of mouse and allow a reduced risk of unnecessary handling of contaminated material when wards request extra (forgotten) analytes. Panels set up with the collaboration of clinicians also improve value for money: by listing only appropriate tests, they allow a reduction in the use of reagents;
  - We describe the ways in which to reorganize staff on the basis of the changes mentioned.
